# The Modulation of Implicit Magnitude on Time Estimates

**DOI:** 10.1371/journal.pone.0046471

**Published:** 2012-10-15

**Authors:** Qingxia Ma, Zhen Yang, Zhijie Zhang

**Affiliations:** 1 College of Education, Hebei Normal University, Shijiazhuang, Hebei, People's Republic of China; 2 Department of Psychology, University of New Mexico, Albuquerque, New Mexico, United States of America; Duke University, United States of America

## Abstract

Studies in time and quantity have shown that explicit magnitude (e.g. Arabic numerals, luminance, or size) modulates time estimates with smaller magnitude biasing the judgment of time towards underestimation and larger magnitude towards overestimation. However, few studies have examined the effect of implicit magnitude on time estimates. The current study used a duration estimation task to investigate the effects of implicit magnitude on time estimation in three experiments. During the duration estimation task, the target words named objects of various lengths (Experiment 1), weights (Experiment 2) and volumes (Experiment 3) were presented on the screen and participants were asked to reproduce the amount of time the words remained on the screen via button presses. Results indicated that the time estimates were modulated by the implicit magnitude of the word's referent with words named objects of smaller magnitude (shorter, lighter, or smaller) being judged to last a shorter time, and words named objects of greater magnitude (longer, heavier, or bigger) being judged to last a longer time. These findings were consistent with previous studies examining the effect of implicit spatial length on time estimates. More importantly, current results extended the implicit magnitude of length to the implicit magnitude of weight and volume and demonstrated a functional interaction between time and implicit magnitude in all three aspects of quantity, suggesting a common generalized magnitude system. These results provided new evidence to support a theory of magnitude (ATOM).

## Introduction

Space, time, and quantity are essential dimensions of human cognitive processes which provide important information about the external environment and contribute to our behaviors in everyday life.. The close relationship among space, time, and quantity has been supported by the behavioral and neuroimaging literature [1], [2], [3], [4]. For example, the mere presentation of task-irrelevant Arabic numbers can influence participants' performance in a duration judgment task with an underestimation bias associated with small numbers and overestimation associated with large numbers [5]. This systematic bias of quantity on time was also observed in imaginative time bisection [6]. Studies on space and time have shown that longer time was associated with lines with longer spatial length [7]. Additionally, spatial position of stimulus could also affect duration judgment with stimuli presented in the left space biasing the judgment of time towards underestimation and stimuli presented in the right towards overestimation [8].

Based on the empirical evidence, Walsh's proposed A Theory of Magnitude (ATOM) [9] and summarized that time, space and quantity were linked to a single coordinative system which represents the magnitude of these dimensions commonly. According to this theory, all dimensions can be characterized by more than and less than, regardless of whether the comparison involves temporal durations, physical distances, or number magnitude. This common representational system was primarily supported by behavioral studies on interference effect in which the magnitude of different dimensions expressed as flashing dots [10], number of dots, size of open squares, luminance of solid squares, or numeric value of digits [11], line length [7], luminance [12], [13] and weights [14] are inter-correlated with each other. This theory was further supported by neuropsychological and brain imaging evidence showing that the inferior parietal cortex was commonly activated during the processing of temporal, spatial and number information in healthy participants [15], [16], [17] and that the co-occurrence of spatial and temporal deficits was observed in patients with parietal lesions [2], [18].

Within the framework of ATOM, the effect of implicit spatial and quantitative magnitude on temporal estimation has been studied extensively. However, it is unknown whether a new variable, implicit magnitude of semantics, would have similar effect on temporal estimation. The semantics of word contains implicit quantity information and the quantity feature is one of the indexes used for semantics judgment. Berent and colleagues [19] demonstrated that when the number of words was congruent with their morphological number, participants' response time was shorter compared with when they are incongruent. These results suggested that the extraction of quantitative information from words was an automatic representation. In a lexical decision task, participants were asked to make a lexical decision on concrete nouns denoting either big or small objects. Results showed that the reaction time was faster when the words encoding a big object compared with a small object, suggesting that semantic size is automatically activated when reading a word [20]. Bottini and Casasanto [21] have also demonstrated that implicit spatial length modulates time estimates, although variations in the duration of the event nouns' referents had no effect on the judgments of the words' spatial length.

The current study extended the implicit magnitude of semantics from length to weight and volume and investigated the effects of the implicit magnitude information encoded in object nouns on time estimation in three experiments (Experiment 1: length; Experiment 2: weight; Experiment 3: volume). According to ATOM, internally generated representations of magnitude may modulate estimates of duration across dimensions. Thus, we hypothesized that the meanings of words would be activated (voluntarily or involuntarily) while participants read the words, and semantics of an objects noun would interference with participants' performance in a duration reproduction task.

## Methods and Results

### Materials

The words used in Experiment 1 were similar to those used by Bottini and colleagues [21]. Words used in Experiment 2 and 3 were generated through brainstorm by the principle investigator and two psychological graduate students. A total of 20 words were generated for each experiment. In a pilot study, three hundred undergraduate students were then asked to rate the feature of these words (most salient in lengths, weights, or volumes) and rank the words with the same feature/dimension in ascending order. Selection rates of these words were computed. The top 8 words within each dimension were selected as the final target words.

Informed consent was obtained from all participants according to institutional guidelines at Hebei Normal Unversity.

### Experiment 1

The aim of the first experiment is to replicate the previous study conducted by Bottini and and Casasanto [21] in which the implicit spatial length has been shown to modulate time estimates.

A total of thirty healthy participants (15 males and 15 females, age range: 27–29 years) participated in Experiment 1. Participants were with normal or corrected to normal vision. Participants were positioned 60 centimeters from a computer monitor configured to a refresh rate of 75 Hz. Chinese nouns (36-point Courier New) naming 8 concrete objects with different length (ranging from short to long) were presented on a computer monitor (resolution = 800×600 pixels) for varying durations (varying from 2000 to 6000 ms in 500 ms increments). English equivalents of these nouns were listed in order of length: matches, cigarette, pencil, chopstick, tubes, javelin, horizontal bar, and flagpole. Each word was presented 9 times in various durations, resulting in a total of 72 trials throughout the experiment.

During the experiment, words were presented one at a time. Immediately after each word, an “hourglass" icon appeared at the center of the screen and served as a cue to indicate that it is ready for the participants to reproduce the amount of time during which the word remained on the screen. To reproduce the duration, participants pressed “D" on the keyboard, waited the appropriate amount of time, and then pressed “K" on the keyboard. The two button presses indicated the beginning and the end of the temporal interval, respectively.

After the behavioral testing, a two-part post-test was included. In the first part, participants were asked to estimate familiarity degree (i.e. rating how familiar an object is to the participant with a five-point rating scale) and association degree (i.e. rating how easily each word elicited mental images with a five-point rating scale) of the target words' referents in this experiment. In the second part, participants were asked to estimate the typical magnitude (length) of the target words' referents using an appropriate unit of measurement. To test whether participants differed in familiarity and association in these words, two one-way repeated-measures ANOVA was performed on these two measures, separately. No main effects of discrepancy were found in either measures, suggesting that familiarity degree and association degree were not contributing to the word judgments. The mean and standard deviation of these two measures were listed in Tables 1 and 2.

**Table 1:** The mean and standard deviation of the familiarity degree and association degree rating in Experiment 1.

Word	Familiarity Degree		Association Degree	
	*M*	*SD*	*M*	*SD*
matches	3.71	1.27	2.18	0.94
cigarette	3.29	1.12	2.46	1.32
pencil	3.79	1.23	2.39	0.99
chopstick	4.50	1.04	2.29	1.05
tube	3.82	1.22	2.14	0.76
javelin	2.68	1.31	2.00	0.98
horizontal bar	3.07	1.41	2.32	1.22
flagpole	3.54	1.14	2.11	0.83

Note: M = mean; SD = standard deviation.

**Table 2:** The mean and standard deviation of the estimated length and the number of strokes for the target words selected for Experiment 1.

Word	Length Estimation (meter)		The Number of Strokes
	*M*	*SD*	
matches	0.04	0.01	14
cigarette	0.10	0.14	19
pencil	0.19	0.11	20
chopstick	0.21	0.07	16
tube	1.15	0.44	20
javelin	1.70	0.87	17
horizontal bar	2.27	0.61	15
flagpole	11.14	5.67	21

Note: M = mean; SD = standard deviation.

Three participants were removed from further analyses. One is missing the post-test data due to misunderstanding the experimental instruction.. The other two were excluded due to poor behavioral performance (i.e. duration reproduction concentrated in less than 2000 milliseconds), leaving a total of 27 participants for final analysis.

**Figure 1:**
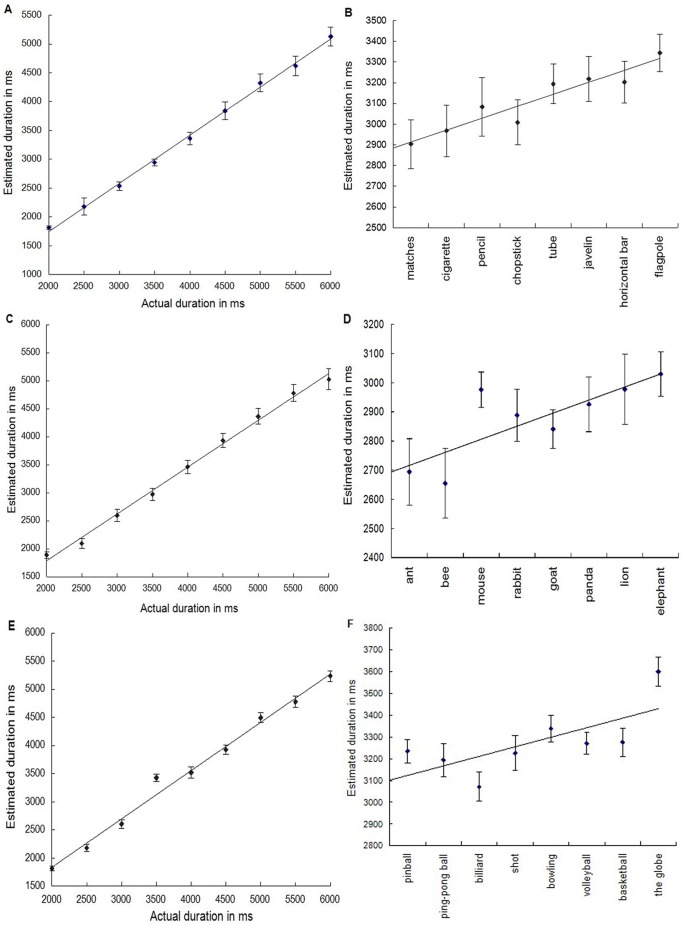
This figure shows the results of Experiment 1 (length: a,b), Experiment 2 (weight: c,d), and Experiment 3 (volume: e,f). In Panels a, c, and e, the estimated duration in millisecond (ms) was plotted as a function of actual duration in millisecond. In Panels b, d, and f, the estimated duration was plotted as a function of the implicit magnitude. Error bars correspond to the standard error of the mean.

To identify the relationship between duration estimates and implicit magnitude, three linear regression analyses similar to what was used by Casasanto and Boroditsky [21] and Lu and colleagues [14] were conducted. Specifically, the actual duration of the stimuli was entered as a regressor to predict participants' subjective duration estimation in the first regression analysis. Overall, mean effect of actual duration on estimated duration was significant(y = 0.84×+77.54, df = 7, r2 = 0.99, p<0.0001. Fig. 1A), suggesting duration estimation for target words were highly accurate. Target words were then rank-ordered according to the typical lengths of their referents. This ranking was confirmed by participants' post-test length estimation. The second regression analysis is on ranked lengths and estimated duration. Results indicated that estimates of duration was affected by implicit lengths (y = 57.75×+2855.37, df = 6, r2 = 0.90, p<0.01, Fig. 1B). The third regression analysis was performed using participants' post-test ratings of the typical length of each target word's referent to predict the duration estimates. Ratings for each target item were averaged across all subjects, and the average length estimates in meter were transformed by a base 10 logarithm due to violation of assumptions of normality and heterogeneity of variance of the raw data. Results indicated a significant effect of implicit length on duration estimation(y = 179.96×+2802.64, df = 6, r2 = 0.86, p<0.01), suggesting that time estimates was associated with implicit lengths of words In addition, no significant correlation between the number of Chinese stroke and the mean of duration estimation was observed, suggesting that the number of Chinese stroke did not contribute significantly to the duration estimation (r(Spearman's rho) = 0.20, p>0.05).

### Experiment 2

Experiment 2 examined whether the implicit weight of a word's referent can modulate estimates of the duration during which the target word was presented.

Twenty-five subjects (15 males and 10 females, age range: 23–26 years) participated in this experiment. Participants performed the same task as in Experiment 1, with the exception that the concrete nouns referred to the weight of an object instead of length. English equivalents of these nouns were listed here in order of increased weight: ant, bee, mouse, rabbit, goat, panda, lion, and elephant.

The data analysis procedure was also identical to Experiment 1. Specifically, a repeated-measures ANOVA was performed on familiarity degree and association degree. Again, results indicated a non-significant main effect of discrepancy in either measure. The mean and standard deviation of these two measures were listed in Tables 3 and 4.

**Table 3:** The mean and standard deviation of the familiarity degree and association degree rating in Experiment 2.

Word	Familiarity Degree		Association Degree	
	*M*	*SD*	*M*	*SD*
ant	4.13	1.35	2.40	1.12
bee	3.86	1.06	2.26	0.96
mouse	3.33	1.49	2.13	1.12
rabbit	3.66	1.54	2.53	1.06
goat	3.33	1.34	2.66	1.44
panda	3.26	1.33	2.53	0.91
lion	2.86	1.18	2.33	1.17
elephant	3.20	1.15	2.53	1.06

Note: M = mean; SD = standard deviation.

**Table 4:** The mean and standard deviation of the estimated weight and the number of strokes for the target words selected for Experiment 2.

Word	Weight Estimation (kg)		The Number of Strokes
	*M*	*SD*	
ant	0.01	0.02	18
bee	0.02	0.05	27
mouse	0.47	0.33	19
rabbit	2.25	1.89	11
goat	57.21	25.45	9
panda	125	125.63	25
lion	188.54	206.91	12
elephant	838.5	659.46	14

Note: M = mean; SD = standard deviation; kg = kilogram.

Two subjects' post-test data was missing due to misunderstanding experiment's instruction and both of them were excluded from further analysis, leaving a final sample of 23 subjects. Linear regression results indicated that duration estimates for target words were highly accurate (mean effect of actual duration on estimated duration: y = 0.84×+112.63, *df* = 7, *r^2^* = 0.99, *p*<0.0001, Fig. 1C). Target words were rank-ordered according to the typical weight of their referents, the significant correlation between ranked weights and duration estimates indicated that the estimates of duration was modulated by the implicit weights (y = 44.84×+2671.7, df = 6, r 2 = 0.65, p<0.05, Fig. 1D). In addition, participants' post-test ratings of the averaged logarithm transformed weight estimates (measured in kilogram) were used as a predictor of the averaged duration of each target word's referent. Results indicated a highly significant effect of implicit weight on duration estimation(y = 57.48×+2662.43, *df* = 6, *r^2^* = 0.63, *p*<0.05) with words named of objects in lighter magnitude being judged as lasting for shorter time, and words named of objects in heavier magnitude as lasting for longer time. Finally, the correlation between the stoke number of Chinese characters and the mean of duration estimates was again not significant (*r*_(Spearman's rho)_ = 0.48,*p*>0.05).

### Experiment 3

Experiment 3 examined whether the implicit volume of a word's referent can modulate estimates of the duration during which the target word was presented.

Thirty-one subjects (15 males and 16 females, age range: 21–26 years) participated in this experiment. Participants performed the same task as in Experiment 1, with the exception that the concrete nouns referred to the volume of an object instead of length. English equivalents of these nouns were listed here in order of increasing volume: pinball, ping-pong ball, billiard, shot, bowling, volleyball, basketball, the globe.

The data analysis procedure was also identical to Experiment 1. Two repeated-measures ANOVAs were performed on familiarity degree and association degree. The statistical results showed no main effect of discrepancy on both measures. The mean and standard deviation of familiarity degree, association degree and estimated volume were listed in Tables 5 and 6.

**Table 5:** The mean and standard deviation of the familiarity degree and association degree rating in Experiment 3.

Word	Familiarity Degree		Association Degree	
	*M*	*SD*	*M*	*SD*
pinball	2.93	1.06	1.85	1.35
ping-pong ball	3.31	0.94	2.14	0.91
billiard	2.75	0.85	1.95	1.32
shot	2.37	0.71	1.57	0.97
bowling	2.62	0.95	1.67	1.11
volleyball	2.93	0.99	2.19	1.08
basketball	3.06	1.06	2.38	1.20
the globe	3.18	0.98	2.04	1.16

Note: M = mean; SD = standard deviation.

**Table 6:** The mean and standard deviation of the estimated volume and the number of strokes for the target words selected for Experiment 3.

Word	Volume Estimation (circumference:cm)		The Number of Strokes
	*M*	*SD*	
pinball	7.41	7.77	22
ping-pong ball	11.32	4.29	23
billiard	19.19	8.96	16
shot	40.72	23.60	21
bowling	55.78	29.34	33
volleyball	71	21.05	22
basketball	82.78	22.34	27

Note: M = mean; SD = standard deviation; cm = centimeter.

Three subjects were removed from final analysis due to poor time estimation performance. Similar regression analysis with participants' duration estimates as a function of the actual duration of the stimuli indicated that duration estimates for target words were highly accurate (mean effect of actual duration on estimated duration: y = 0.86×+122.48,*df* = 7, *r^2^* = 0.99, *p*<0.0001. Fig. 1E). The correlation analysis between the typical volumes of their referents and the duration estimation indicated that implicit volumes affected estimates of duration (y = 43.83×+3079.00, df = 6, r2 = 0.50, p<0.05, Fig. 1F). Finally, the regression analysis on the post-test ratings of the typical duration of each target word's referent revealed that a highly significant effect of implicit volume on duration estimation (y = 176.94×+2968.75, *df* = 5, *r^2^* = 0.75, *p*<0.05) with words that named smaller volume objects being judged to last a shorter time, and words that named bigger volume objects to last a longer time. In addition, The correlation analysis between the number of Chinese stroke and the mean of duration estimates was not significant (*r*_(Spearman's rho)_ = 0.35, *p*>0.05).

## Discussion

The current study examined whether implicit magnitude of length, weight, and volume encoded in concrete object nouns can modulate estimates of time in three experiments using a duration reproduction task (Experiment 1: length; Experiment 2: weight; Experiment 3: volume). Our key finding was that the duration reproduction was compressed or expanded depending on the implicit length, weight, and volume of the word's referent. Specifically, words that named shorter/lighter/smaller objects were judged to last for shorter time, and words that named longer/heavier/bigger objects were judged to last for longer time. These results replicated previous study showing that the implicit spatial length modulates time estimates [21] and extended previous study to Chinese words. More importantly, the current study extended the implicit magnitude of semantics from length to weight and volume and found that the implicit magnitude of these dimensions encoded in object nouns also exert an effect on time estimation.

Word frequency, meaning ambiguity, semantic range, and other meaning hybrid were known to have an impact on word judgments [22]. One advantage of the current experimental design was that potential confounding factors such as complexity or familiarity were controlled [23], [24], as no statistically significant effect of discrepancy was observed in measures indicating familiarity degree and association degree. Furthermore, the target words were selected in a pilot study. This procedure was used to control for the salience of the word's characteristics so that length/weight/volume component of the word's referent was the most salient characteristic encoded in these words. The observed pattern could also be a result of unintended features of the stimulus words. For example, it is possible that duration estimates were influenced by the number of Chinese stroke, rather than implicit length/weight/volume. However, a non-significant correlation between the number of Chinese stroke and the mean of duration estimation in all three experiments allowed us to rule out this possibility.

The results on duration data indicated that the actual duration of object nouns accounted for 99% of the variance in duration judgments, suggesting that duration reproduction for the time the target words remained on the screen were highly accurate Additionally, the regression analysis between implicit magnitude and time showed that the quantity of words' referents exerted order variations, which was corresponding to variations of duration. Taken together, the current results suggested that the implicit magnitude biased duration estimation.

One possible explanation of this cross-dimensional interaction is the processing mechanism of explicit magnitude. Previous studies have suggested that semantics implied quantity information was activated automatically during the processing of semantics [25], [26]. When Chinese words were presented, processing of words' referents may activate the magnitude information. In time cognition, implicit magnitude has been shown to have similar processing mechanism to explicit magnitude. That is, when participants were asked to estimate duration, they may refer to reality quantity of objects to reproduce the duration although the magnitude is implicit. Participants kept track of implicit magnitude even when time was the target. This is consistent with previous findings reporting that the numerical information interfered with subjects' ability to discriminate duration.

In accordance with ATOM, a common magnitude system was activated by implicit quantity and time magnitude input, although the implicit quantity magnitude was irrelevant to the task. The magnitude of one dimension was transformed and exerted effect on the processing of the magnitude of the other dimension. The current results lend further support to ATOM and extended the cross-dimensional interference effect to other implicit quantities, such as weight and volume. What is worth mentioning is that the current study only allowed us to examine the effect of implicit magnitude on duration estimates but not the effect of duration estimates on implicit magnitude. As the interactions between different dimensions of the magnitude system may not be symmetrical [27], [28], future studies should examine whether time information modulates the processing of implicit quantity in words.

## References

[1] Bueti D, Walsh V (2009) The parietal cortex and the representation of time, space, number and other magnitudes. Philosophical Transactions of the Royal Society B Biological Sciences 364: 1831–1840. 10.1098/rstb.2009.0028 PMC2685826 19487186

[2] Cappelletti M, Freeman ED, Cipolotti L (2009) Dissociations and interactions between time, numerosity and space processing. Neuropsychologia 47 13 2732–2748. 19501604 10.1016/j.neuropsychologia.2009.05.024 PMC2796173

[3] Oliveri M, Koch D (2009) Spatial-temporal interactions in human brain. Experimental Brain Research 195: 498–497. 10.1007/s00221-009-1834-1 19458940

[4] Xuan B, Chen XC, He S, Zhang DR (2009) Numerical magnitude modulates temporal comparison: an ERP study. Brain Research 1269: 135–142. 19306851 10.1016/j.brainres.2009.03.016

[5] Oliveri M, Vicario CM, Salerno S, Koch G, Turriziani P, et al (2008) Perceiving numbers alters time perception. Neuroscience Letters 438: 308–311. 18486340 10.1016/j.neulet.2008.04.051

[6] Vicario CM (2007) Selective bias in temporal bisection task by number exposition. Nature Precedings, Available from Nature Precedings. Available: http://dx.doi.org/10.1038/npre.2007.1479.1.

[7] Cssasanto D, Boroditsky L (2008) Time in mind:Using space to think about time. Cognition 106: 579–593. 17509553 10.1016/j.cognition.2007.03.004

[8] Vicario CM, Pecoraro P, Turriziani P, Koch G, Caltagirone C, et al (2008) Relativistic Compression and Expansion of Experiential Time in the Left and Right Space. PLoS ONE 3 3 e1716 doi:10.1371/journal.pone.0001716. 18320037 10.1371/journal.pone.0001716 PMC2248621

[9] Walsh V (2003) A theory of magnitude: Common cortical metrics of time, space and quantity. Trends in Cognitive Sciences 7: 483–488. 14585444 10.1016/j.tics.2003.09.002

[10] Dormal V, Seron X, Pesenti M (2006) Numerosity-duration interference: a Stroop experiment. Acta Psychologica 121 2 109–124. 16095549 10.1016/j.actpsy.2005.06.003

[11] Xuan B, Zhang D, He S, Chen X (2007) Larger stimuli are judged to last longer. Journal of Vision 7: 1–5. 10.1167/7.10.2 17997671

[12] Cohen Kadosh R, Henik A (2006) A Common Representation for Semantic and Physical Properties: A Cognitive-Anatomical Approach. Experimental Psychology 53 2 87–94. 16909932 10.1027/1618-3169.53.2.87

[13] Cohe Kadosh R, Lammertyn J, Izard V (2008) Are numbers special? An overview of chronometric, neuroimaging, developmental and comparative studies of magnitude representation. Progress in Neurobiology 84 2 132–147. 18155348 10.1016/j.pneurobio.2007.11.001

[14] Lu A, Hodges B, Zhang J, Zhang XJ (2009) Contextual effects on number-time interaction. Cognition 113: 117–122. 19640515 10.1016/j.cognition.2009.07.001

[15] Conson M, Cinque F, Barbarulo AM, Trojano L (2008) A common processing system for duration, order and spatial information: evidence from a time estimation task. Experimental Brain Reseach 187: 267–274. 10.1007/s00221-008-1300-5 18265966

[16] Vicario CM, Caltagirone C, Oliveri M (2007) Optokinetic stimulation affects temporal estimation in healthyhumans. Brain and Cognition 64: 68–73. 17397979 10.1016/j.bandc.2006.12.002

[17] Srinivasan M, Carey S (2010) The long and the short of it: on the nature and origin of functional overlap between representations of space and time. Cognition 116: 217–241. 20537324 10.1016/j.cognition.2010.05.005 PMC2900540

[18] Basso G, Nichelli P, Frassinetti F, di Pellegrino G (1996) Time perception in a neglected space. Neuroreport 7: 2111–2114. 8930969 10.1097/00001756-199609020-00009

[19] Berent I, Pinker S, Tzelgov J, Bibi U, Goldfar L (2005) Computation of semantic number from morphological information. Journal of Memory and Language 53: 342–358.

[20] Sereno SC, O'Donnell PJ, Margaret E, Sereno ME (2009) Size matters: Bigger is faster. The Quarterly Journal of Experimental Psychology 62: 1115–1122. 19142830 10.1080/17470210802618900

[21] Bottini R, Casasanto D (2010) Implicit spatial length modulates time estimates, but not vice versa. Proceedings of the 32nd Annual Conference of the Cognitive Science Society 1348–1353.

[22] Rayner K, Sereno SC (1994) Eye movements and reading: Psycholiniguistic studies. In: Gernsbacher MA, editor. Handbook of psycholinguistics. New York: Academic Press. pp: 57–81.

[23] Schiffman HR, Bobko DJ (1974) Effects of stimulus complexity on the perception of brief temporal intervals. Journal of Experimental Psychology 103: 156–159. 4418727 10.1037/h0036794

[24] Schiffman HR, Bobko DJ (1977) The role of number and familiarity of stimuli in the perception of brief temporal intervals. American Journal of Psychology 90: 85–93. 871180

[25] Pavio A (1975) Perceptual comparisons through the mind's eye. Memory & Cognition 3: 635–647. 24203905 10.3758/BF03198229

[26] Rubinsten O, Henik A (2002) Is an ant larger than a lion? Acta Psychologica 111: 141–154. 12102118 10.1016/s0001-6918(02)00047-1

[27] Droit-Volet S, Clement A, Fayol M (2003) Time and number discrimination in a bisection task with a sequence of stimuli: A developmental approach. Journal Experimental Child Psychology 84: 63–76. 10.1016/s0022-0965(02)00180-7 12553918

[28] Roitman JD, Brannon EM, Andrews JR, Platt ML (2007) Nonverbal representation of time and number in adults. Acta Psychologica 124: 296–318. 16759623 10.1016/j.actpsy.2006.03.008

